# MRI versus CT for the detection of pulmonary nodules: A meta-analysis

**DOI:** 10.1097/MD.0000000000027270

**Published:** 2021-10-22

**Authors:** Hui Liu, Rihui Chen, Chao Tong, Xian-Wen Liang

**Affiliations:** Central South University Xiangya School of Medicine Affiliated Haikou Hospital, Haikou, China.

**Keywords:** computed tomography, magnetic resonance imaging, meta-analysis, pulmonary nodules

## Abstract

**Background:**

Computed tomography (CT) is the current gold standard for the detection of pulmonary nodules but has high radiation burden. In contrast, many radiologists tried to use magnetic resonance imaging (MRI) to replace CT because MRI has no radiation burden associated. Due to the lack of high-level evidence of comparison of the diagnostic accuracy of MRI versus CT for detecting pulmonary nodules, it is unknown whether CT can be replaced successfully by MRI. Therefore, the aim of this study was to compare the diagnostic accuracy of MRI versus CT for detecting pulmonary nodules.

**Methods:**

Electronic databases PubMed, EmBase, and Cochrane Library were systematically searched from their inception to September 2017 to identify studies in which CT/MRI was used to diagnose pulmonary nodules. According to true positive, true negative, false negative, and false positive extracted from the included studies, we calculate the pooled sensitivity, specificity, positive likelihood ratio (PLR), negative likelihood ratio (NLR), and area under the curve (AUC) using Stata version 14.0 software (STATA Corp, TX).

**Results:**

A total of 8 studies involving a total of 653 individuals were included. The pooled sensitivity, specificity, PLR, NLR, and AUC were 0.91 (95% confidence interval [CI]: 0.80–0.96), 0.76 (95%CI: 0.58–0.87), 3.72 (95%CI: 2.05–6.76), 0.12 (95%CI: 0.06–0.27), and 0.91 (95%CI: 0.88–0.93) for MRI respectively, while the pooled sensitivity, specificity, PLR, NLR, and AUC for CT were 1.00 (95%CI: 0.95–1.00), 0.99 (95%CI: 0.78–1.00), 79.35 (95%CI: 3.68–1711.06), 0.00 (95%CI: 0.00–0.06), and 1.00 (95%CI: 0.99–1.00), respectively. Further, we compared the diagnostic accuracy of CT versus MRI and found that compared with MRI, CT shows statistically higher sensitivity (odds ratio [OR] for MRI vs CT: 0.91; 95%CI: 0.85–0.98; *P* value .010), specificity (OR: 0.82; 95%CI: 0.69–0.97; *P* value .019), PLR (OR: 0.29; 95%CI: 0.10–0.83; *P* value 0.02), AUC (OR: 0.91; 95%CI: 0.89–0.94; *P* value < .001), and lower NLR (OR: 8.72; 95%CI: 1.57–48.56; *P* value .013).

**Conclusion:**

Our study suggested both CT and MRI have a high diagnostic accuracy in diagnosing pulmonary nodules, while CT was superior to MRI in sensitivity, specificity, PLR, NLR, and AUC, indicating that in terms of the currently available evidence, MRI could not replace CT in diagnosing pulmonary nodules.

## 1 Introduction

Pulmonary nodules are a common disease including viral pulmonary nodule,^[32]^ bacterial pulmonary nodule, and malignant pulmonary nodule, which can result in several complications involving pulmonary fibrosis and pulmonary hypertension.^[33]^ A solitary pulmonary nodule is widely encountered in multi detector computed tomography (CT),^[1]^ and the screening of a high-risk peoples with low-dose CT was associated with 20% reduction of lung cancer mortality.^[2]^ Nevertheless, although frequent use of CT in screening and follow-up to observe the growth rate of pulmonary nodules is acceptable,^[3,4]^ it was associated with considerable cumulative radiation exposure, which could stimulate the progression of cancer even most of individuals were diagnosis with benign pulmonary disease.^[5–7]^ Therefore, it is necessary to find additional alternative technique without radiation exposure for detecting pulmonary nodules.

Pulmonary magnetic resonance imaging (MRI) has been introduced and increased using in parenchymal evaluation, including bronchopulmonary dysplasia, cystic fibrosis, cardio-pulmonary vascular abnormalities, and intra-thoracic tumors, and without using of ionizing radiation.^[8,9]^ The value of MRI in patients with pulmonary nodules have already illustrated in several meta-analyses.^[10–12]^ Jiang et al^[10]^ pooled 12 studies with 524 malignant and 284 benign nodules and indicated the diagnosis parameters for discrimination of benign from malignant pulmonary nodules were relative higher (sensitivity: 0.95; specificity: 0.87; positive likelihood ratio [PLR]: 7.60; negative likelihood ratio [NLR]: 0.06; and area under receiver operating characteristic [ROC] curves: 0.94), while this study could not provide the direct comparison with the diagnostic value of CT. Similar limitations are detected in the study conducted by Li et al,^[11]^ which just provide the diagnosis parameters for MRI detection of malignant pulmonary nodules and masses. Further, these studies main focused on discriminating benign and malignant pulmonary nodules and not studied the detecting accuracy rate for pulmonary nodules. Cronin et al^[12]^ evaluate the diagnostic value of CT, MRI, positron emission tomography, and single photon emission CT for the evaluation of solitary pulmonary nodule, while the comparisons of the diagnostic value directly were not calculated and provided relative synthetic results. Clarifying the diagnostic value of alternative technique namely MRI is particularly important for detecting pulmonary nodules, as it has not been definitively determined. Therefore, we attempted examination of the available studies to compare the diagnostic value between MRI and CT for detecting pulmonary nodules directly. The results of our study will provide evidence-based summaries for whether CT can be replaced successfully by MRI for detection of pulmonary nodules, which may assist the clinicians in making decisions about the selection of diagnostic methods.

## 2 Methods

### 2.1 Data sources, search strategy, and selection criteria

The Research Ethics Committee of Central South University Xiangya School of Medicine Affiliated Haikou Hospital approved this study. This review was conducted and reported according to the Preferred Reporting Items for Systematic Reviews and Meta-Analysis Statement issued in 2009.^[13]^ The study compared the diagnostic value between MRI and CT for detecting pulmonary nodules were eligible for inclusion in this study, and no restriction was placed on publication status (published, in press, or in progress). Further, we restricted the study published in English. We searched the PubMed, EmBase, and Cochrane Library electronic databases for articles published with inception to September 2017 and used ((“nodules” or “nodule”) AND (“lung” or “pulmonary”)) AND ((“computed tomography” or “CT”) and (“Magnetic Resonance Imaging” or “MRI”)) AND “human” AND “English” as the search terms. Manual searches of reference lists from relevant studies were performed to identify any potential eligible studies. The study topic, diagnostic tool, control, patient's status, and the outcomes reported were employed to select potential relevant studies.

The literature search was independently undertaken by 2 authors using a standardized approach. Any inconsistencies between these 2 authors were settled by the primary author until a consensus was reached. The study was eligible for inclusion if the following criteria were met: patients with pulmonary lesions or with high risk of pulmonary nodules; patients with MRI and CT for detecting pulmonary nodules; and the study provided true positive, false positive, false negative, true negative for MRI, and CT diagnostic results.

### 2.2 Data collection and quality assessment

The data collected included the first author's name, publication year, country, sample size, mean age, and number of men and women, true and false positive and negative for MRI and CT, respectively. The Quality Assessment of Diagnostic Accuracy Studies (QUADAS), which is quite comprehensive and has been partially validated for evaluating the quality of studies in diagnosis meta-analysis, was used to evaluate methodological quality.^[14,15]^ The QUADAS is based on the following sub-scales: representative patient spectrum, reporting of selection criteria, reference standard, absence of disease progression bias, absence of partial verification bias, absence of differential verification bias, absence of incorporation bias, description of index text execution, description of reference standard execution, reference standard blinded, index test blinded, absence of clinical review bias, reporting of uninterpretable/intermediate results, and withdrawal. Each of sub-scale was regarded as “yes”, “no”, or “NA”. The data extraction and quality assessment were conducted independently by 2 authors. Information was examined and adjudicated independently by an additional author referring to the original studies.

### 2.3 Statistical analysis

We calculated the summary sensitivity, specificity, PLR, NLR, and corresponding 95% confidence intervals (CIs) by bivariate random effects for MRI and CT respectively on the basis of true positive, false positive, false negative, and true negative in each study.^[16]^ Further, the summary receiver operating characteristic curve and the area under the curve (AUC) for MRI and CT respectively by using the hierarchical regression model.^[17]^ In addition, the diagnosis parameters with corresponding 95%CI were abstracted for MRI and CT in each study, and the summary ratio between MRI and CT and 95% CIs for were sensitivity, specificity, PLR, NLR by using the random-effects model.^[18]^

Heterogeneity between studies was investigated by using the Q statistic, and we considered *P* values < .10 as indicative of significant heterogeneity.^[19,20]^ Subgroup analyses were conducted for sensitivity, specificity, PLR, NLR on the basis of publication year, country, and mean age. Deeks asymmetry test for MRI and CT were calculated and presented as funnel plots.^[21]^ All reported *P* values are 2-sided, and *P* values < .05 were considered statistically significant for all included studies. Statistical analyses were performed using STATA software (version 10.0; Stata Corporation, College Station, TX).

## 3 Results

**Figure 1:**
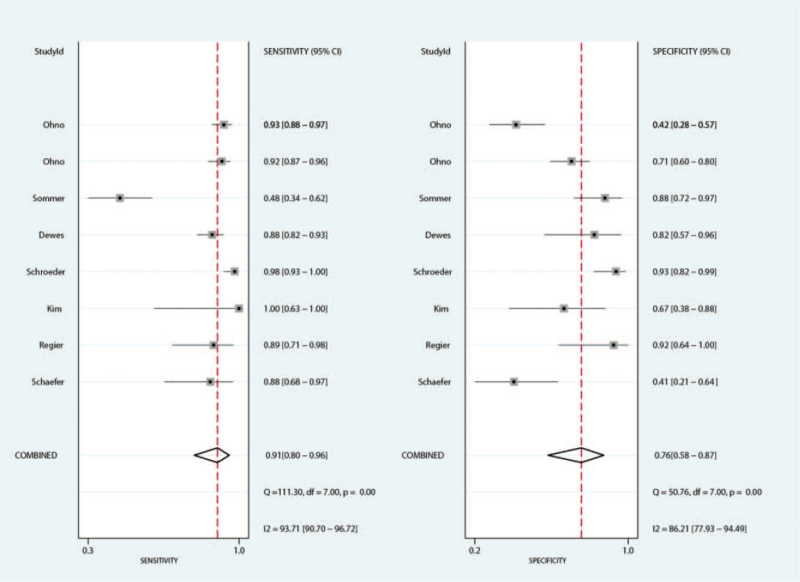
Flow diagram of the literature search and trials selection process.

The results of study-selection process are shown in Figure 1. We identified 631 articles in our initial electronic search, of which 589 were identified after duplicates and irrelevant studies were excluded. A total of 42 potentially eligible studies were selected. After detailed evaluations, 8 prospective studies with a total of 2628 individuals were selected for the final meta-analysis.^[22–29]^ A manual search of the reference lists of these studies did not yield any new eligible studies. The general characteristics of the included studies are presented in Table 1. The assessment outcome of the each study by using QUADAS are listed in Table 2.

**Table 1:** Characteristics of the studies included in the meta-analysis.

						Magnetic resonance imaging				Computed tomography			
Author	Year	Country	N	Mean age	Men/women	True positive	False positive	True negative	False negative	True positive	False positive	True negative	False negative
Schaefer et al^[22]^	2006	Germany	46	61.0	36/10	21	13	9	3	23	13	9	1
Regier et al^[23]^	2011	Germany	20	66.4	10/10	24	1	12	3	27	0	13	0
Kim et al^[24]^	2004	Korea	81	50.0	54/27	8	5	10	0	7	6	9	1
Schroeder et al^[25]^	2005	Germany	30	53.3	19/11	102	3	43	2	102	2	43	3
Dewes et al^[26]^	2016	Germany	54	60.8	27/27	121	3	14	16	137	0	14	0
Sommer et al^[27]^	2014	Germany	49	61.0	31/18	26	4	29	28	54	0	33	0
Ohno et al^[28]^	2015	Japan	198	75.4	111/87	123	25	60	10	133	0	85	0
Ohno et al^[29]^	2008	Japan	175	72.1	92/83	142	29	21	10	152	0	50	0

**Table 2:** Quality evaluation of the included studies using the QUADAS tool.

	Question about study design characteristic													
Study	Representative patient spectrum	Reporting of selection criteria	Reference standard	Absence of disease progression bias	Absence of partial verification bias	Absence of differential verification bias	Absence of incorporation bias	Description of index text execution	Description of reference standard execution	Reference standard blinded	Index test blinded	Absence of clinical review bias	Reporting of uninterpretable/intermediate results	Withdrawal
Schaefer	Yes	Yes	Yes	Yes	Yes	Yes	Yes	Yes	Yes	No	No	Yes	Yes	Yes
Regier	Yes	Yes	Yes	Yes	Yes	Yes	Yes	Yes	Yes	No	No	Yes	NA	Yes
Kim	Yes	Yes	Yes	Yes	Yes	Yes	Yes	Yes	Yes	No	No	Yes	NA	Yes
Schroeder	Yes	Yes	Yes	Yes	Yes	Yes	Yes	Yes	Yes	No	No	Yes	NA	Yes
Dewes	Yes	Yes	Yes	Yes	Yes	Yes	Yes	Yes	Yes	No	No	Yes	Yes	Yes
Sommer	Yes	Yes	Yes	Yes	Yes	Yes	Yes	Yes	Yes	No	No	Yes	NA	Yes
Ohno	Yes	Yes	Yes	Yes	Yes	Yes	Yes	Yes	Yes	No	No	Yes	NA	Yes
Ohno	Yes	Yes	Yes	Yes	Yes	Yes	Yes	Yes	Yes	No	No	Yes	NA	Yes

**Figure 2:**
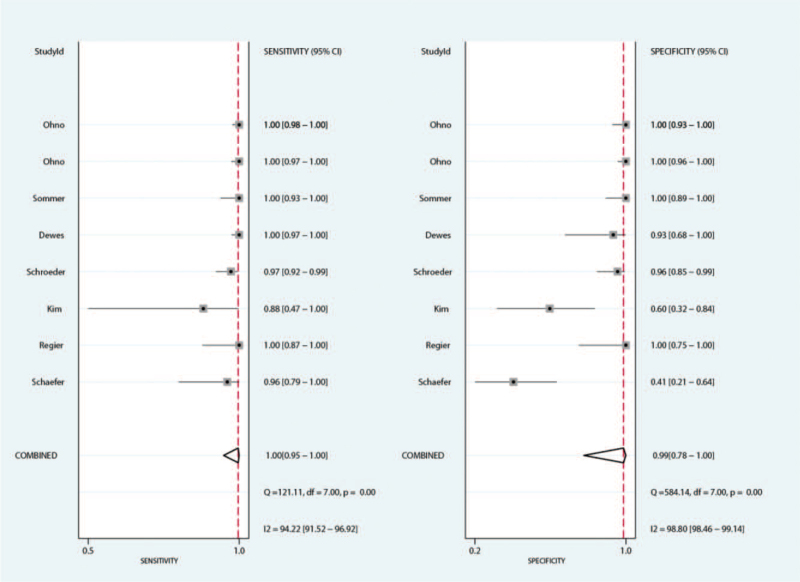
The summary results for sensitivity between MRI and CT. CT = computed tomography, MRI = magnetic resonance imaging.

The summary sensitivity for MRI and CT for detecting pulmonary nodules were 0.91 (95%CI: 0.80–0.96) and 1.00 (95%CI: 0.95–1.00), respectively. We noted MRI with lower sensitivity for detecting pulmonary nodules when compared with CT (ratio between MRI and CT: 0.91; 95%CI: 0.85–0.98; *P* = .010; Fig. 2). Subgroup analyses suggested there was no significant difference for sensitivity between MRI and CT if the study published before 2010, or mean age of included patients <65.0 years (Table 3).

**Table 3:** Subgroup analyses for diagnostic accuracy comparison of MRI versus CT by publication year, country, and mean age.

Outcomes	Group	Gender	Odds ratio between MRI and CT and 95%CI	*P* value	I2 (%)	*P* value for heterogeneity
Sensitivity	Publication year	2010 or after	0.84 (0.75–0.95)	.007	83.0	.001
		Before 2010	0.97 (0.92–1.03)	.313	35.8	.197
	Country	Europe	0.86 (0.76–0.99)	.033	86.2	<.001
		Asia	0.94 (0.90–0.97)	<.001	0.0	.685
	Mean age	≥65.0	0.93 (0.90–0.97)	<.001	0.0	.875
		<65.0	0.87 (0.75–1.01)	.073	86.3	<.001
Specificity	Publication year	2010 or after	0.83 (0.72–0.95)	.007	43.3	.152
		Before 2010	0.80 (0.48–1.31)	.369	84.2	<.001
	Country	Europe	0.93 (0.86–1.02)	.122	0.0	.928
		Asia	0.66 (0.43–1.03)	.065	79.0	.009
	Mean age	≥65.0	0.67 (0.47–0.95)	.026	83.3	.003
		<65.0	0.94 (0.86–1.03)	.166	0.0	.883
PLR	Publication year	2010 or after	0.12 (0.02–0.65)	.014	45.1	.141
		Before 2010	0.57 (0.19–1.70)	.310	70.7	.017
	Country	Europe	0.78 (0.49–1.26)	.311	0.0	.424
		Asia	0.08 (0.00–2.31)	.143	89.2	<.001
	Mean age	≥65.0	0.04 (0.01–0.23)	<.001	29.7	.241
		<65.0	0.85 (0.51–1.40)	.512	9.6	.352
NLR	Publication year	2010 or after	30.84 (7.69–123.61)	<.001	24.2	.266
		Before 2010	1.90 (0.28–13.03)	.512	55.2	.082
	Country	Europe	7.77 (1.02–59.17)	.048	79.8	.001
		Asia	14.44 (0.20–1019.62)	.219	73.0	.025
	Mean age	≥65.0	22.21 (2.39–206.20)	.006	27.3	.253
		<65.0	4.86 (0.46–51.72)	.190	82.9	<.001

**Figure 3:**
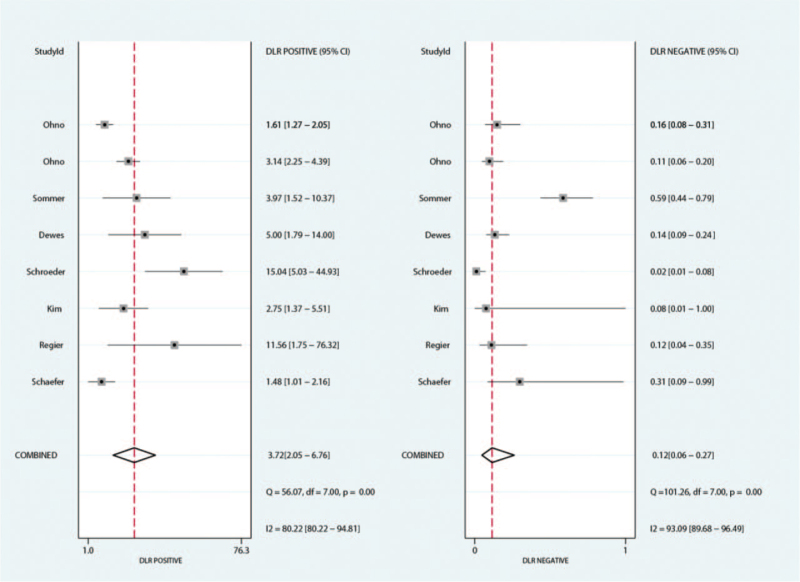
The summary results for specificity between MRI and CT. CT = computed tomography, MRI = magnetic resonance imaging.

The summary specificity for MRI and CT for detecting pulmonary nodules were 0.76 (95%CI: 0.58–0.87) and 0.99 (95%CI: 0.78–1.00), respectively. The summary results suggested MRI with lower specificity for detecting pulmonary nodules when compared with CT specificity (ratio between MRI and CT: 0.82; 95%CI: 0.69–0.97; *P* = .019; Fig. 3). This significantly difference was observed in the study published in 2010 or after, and the mean age of included patients ≥65.0 years (Table 3).

**Figure 4:**
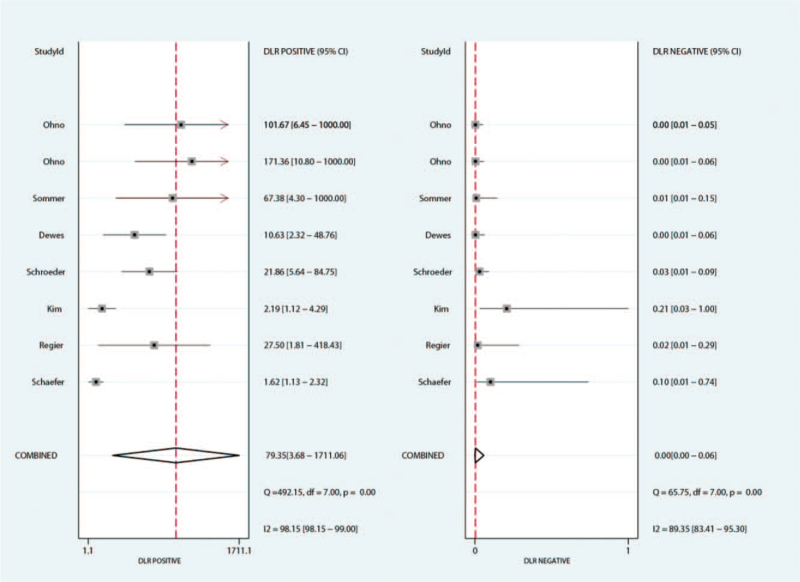
The summary results for PLR between MRI and CT. CT = computed tomography, MRI = magnetic resonance imaging, PLR = positive likelihood ratio.

The summary PLR for MRI and CT for detecting pulmonary nodules were 3.72 (95%CI: 2.05–6.76) and 79.35 (95%CI: 3.68–1711.06), respectively. We noted MRI was associated with lower PLR for detecting pulmonary nodules (ratio between MRI and CT: 0.29; 95%CI: 0.10–0.83; *P* = .020; Fig. 4). The findings of subgroup analysis found the significant difference between MRI and CT for PLR if the study published in 2010 or after, and the mean age of included patients ≥65.0 years (Table 3).

The summary NLR for MRI and CT for detecting pulmonary nodules were 0.12 (95%CI: 0.06–0.27) and 0.00 (95%CI: 0.00–0.06), respectively. The summary results indicated MRI with higher NLR for detecting pulmonary nodules when compared with CT (ratio between MRI and CT: 0.29; 95%CI: 0.10–0.83; *P* = .020). Subgroup analysis suggested the significant difference between MRI and CT was prominent for NLR in study published in 2010 or after, the study conducted in Europe, and the mean age of included patients ≥65.0 years (Table 3).

The summary area under ROC for MRI and CT for detecting pulmonary nodules were 0.91 (95%CI: 0.88–0.93) and 1.00 (95%CI: 0.99–1.00), respectively. We noted MRI was associated with lower diagnostic value for detecting pulmonary nodules as compared with CT (ratio between MRI and CT: 0.91; 95%CI: 0.89–0.94; *P* < .001).

Review of the funnel plots could not rule out the potential for publication bias for MRI and CT for detecting pulmonary nodules. The Deeks asymmetry test results showed no evidence of publication bias for MRI (*P* = .84), and potential evidence of publication bias for CT (*P* = .02).

## 4 Discussion

Our current study compared the diagnostic value of MRI and CT for detecting pulmonary nodules. This comprehensive quantitative study included 653 individuals from 8 published studies with a broad range of populations. The findings from our current meta-analysis indicated MRI has relative higher diagnostic value for detecting pulmonary nodules with in terms of higher sensitivity, specificity, PLR, AUC, and lower NLR. However, when compared with the diagnostic value of CT for detecting pulmonary nodules, MRI was associated with lower sensitivity, specificity, PLR, AUC, and higher NLR, which indicate in terms of the currently available evidence, MRI could not replace CT in diagnosing pulmonary nodules. Next, we performed a subgroup analysis for diagnostic accuracy comparison of MRI versus CT by publication year, country, and mean age, and MRI has even poorer diagnostic accuracy than CT regardless of publication year, country, and mean age. The probable reasons of lower diagnostic accuracy are as follows: on the one hand, the lung is composed primarily of trachea, bronchus, alveolus pulmonis, blood vessels, and lymphatic vessels, and alveolus pulmonis which harbor a significant amount of air is the major component of lung, which indicate low diagnostic accuracy of MRI because MRI is a functional imaging technique based on water diffusivity. On the other hand, there is a sharp density difference between normal tissue and lung lesion which can be accurately detected by CT.

A previous meta-analysis suggested that patients with dynamic contrast-enhanced MRI is valuable for distinguish benign or malignant pulmonary nodules, and especially for discrimination of malignant pulmonary nodules, while the study just reported the summary diagnosis parameters for MRI, and the comparison of MRI versus CT were not available. Further, it is unknown whether the diagnostic value is different in specific subpopulation patients.^[10]^ Li et al^[11]^ conducted a meta-analysis based on 17 studies involving 855 malignant and 322 benign lesions. They indicated MRI is valuable for differentiating malignant from benign pulmonary nodules or masses, and the summary results of diagnostic performance in retrospectively designed studies was higher than prospectively designed studies. The study did not compare MRI and CT for detecting pulmonary nodules directly. Cronin et al^[12]^ reported the summary results of diagnostic performance in CT, MRI, positron emission tomography, and single photon emission CT for detecting solitary pulmonary nodules, respectively. However, the summary results was based on various multiple studies including the study evaluate single diagnostic tool for detecting pulmonary nodules, and the direct comparison of diagnostic performance between MRI and CT for detecting pulmonary nodules were not calculated. Further, subgroup analyses for diagnostic performance based on study or participants characteristics were not evaluated. Therefore, we conducted a meta-analysis to directly compare the diagnostic performance between MRI and CT for detecting pulmonary nodules.

The findings of this study suggested MRI was associated with lower diagnostic performance for detecting pulmonary nodules when compared with CT, whereas numerous studies did not provide the comparison of MRI and CT diagnostic performance. Schaefer et al^[22]^ reported the mean sensitivity, specificity, and accuracy of observers for MRI were 89%, 42%, and 66%, respectively. They concluded no significant difference of accuracy between MRI and CT. The reason for this could be the optimized signal gain using a proton density weighted GE with a short echo time and a low flip angle. Further, the study used ECG-triggering might contribute an important reason for this high accuracy of MRI. Regier et al^[23]^ suggested patients with diffusion-weighted imaging MRI for detection of nodules ≥6 mm with reasonably high sensitivity rates, while the results of false positive decreases the accuracy of MRI as compared with CT. Kim et al^[24]^ reported similar diagnostic performance for differentiation between benign and malignant solitary pulmonary nodule. The reason for this could be different threshold values were correlated with different MRI techniques, which include pulse sequence and imaging acquisition time. Schroeder et al^[25]^ suggested the sensitivity of HASTE MRI was 73% for lesions less than 3 mm, 86.3% for lesions between 3 and 5 mm, 95.7% for lesions between 6 and 10 mm, and 100% for lesions greater than 10 mm. They concluded HASTE MRI could be employed screen pulmonary lesions that are 5 mm and bigger, and the lesions smaller than 5 mm needed to be validated by CT. Dewes et al^[26]^ reported similar findings as compared with the study conducted by Schroeder et al,^[25]^ which used non-contrast Controlled Aliasing In Parallel Imaging Results In Higher Acceleration volumetric interpolated breath-hold examination 3T MRI. Sommer et al^[27]^ suggested MRI has greater valuable for detecting malignant nodules than to benign ones, and should employ to detect early stage lung cancer. The reason for this is due to inherent soft tissue contrast of MRI. The findings of Ohno et al^[28,29]^ reported similar findings contrast with the current meta-analysis, and suggested CT is more sensitive than MRI for detecting solitary pulmonary nodules in routine clinical practice. The possible reason for this could be that time-density and time-signal intensity course curves in wish-in phase were associated with the combination of perfusion (blood flow per unit of tissue), microvascular density (tumor angiogenesis), extracellular space for accumulation of contrast material, and permeability of capillaries, which might contribute an important role for the diagnosis performance of MRI.^[30,31]^

Subgroup analysis suggested publication year, country, and mean age might affect the diagnostic performance between MRI and CT for detecting pulmonary nodules. The possible reason for this could be that the diagnostic techniques are different across the study published years. Further, the diagnostic techniques in Europe and Asia might differ and affect the diagnostic performance for detecting pulmonary nodules. In addition, the mean age of patients might affect the progression of pulmonary nodules, which associated with different size and morphologic features. Finally, although diagnostic performance is different according to published year, country, and mean age of included patients, while these conclusions may be variable since smaller studies were included in such subset. Therefore, we just gave a relative result and provided a synthetic and comprehensive review.

## 5 Conclusion

In conclusion, the results of this meta-analysis indicated MRI has lower diagnostic performance for detecting pulmonary nodules when compared with CT. Further, according to the subgroup analysis results for diagnostic accuracy comparison of MRI versus CT by publication year, country, and mean age, MRI has even poorer diagnostic accuracy than CT regardless of publication year, country, and mean age. Therefore, our studies indicate in terms of the currently available evidence, MRI could not replace CT in diagnosing pulmonary nodules because of poorer diagnostic accuracy.

### 5.1 Limitations

The limitations of our study are as follows: the diagnostic performance based on size of pulmonary nodules of MRI versus CT were not calculated because most of the included studies did not report the diagnostic performance based on size of pulmonary nodules for MRI or CT; different diagnostic techniques in MRI and CT might affect the diagnostic accuracy for detecting pulmonary nodules; in a meta-analysis of published studies, publication bias is an inevitable problem; the meta-analysis is based on study level results but not original data of an individual patient, which restricted us to present a more comprehensive result. Therefore, more future studies are required to prove our conclusions.

## Author contributions

Hui Liu and Xianwen Liang conceived and designed the study. All authors were responsible for data collection, analysis, interpretation, and generation of figures, and Hui Liu, Rihui Chen, and Chao Tong were involved in writing of the paper. Xianwen Liang took part in paper checking and modification, and gave final approval of the manuscript. All authors contributed to the article and approved the submitted version.

**Formal analysis:** Rihui Chen, Chao Tong.

**Methodology:** Chao Tong, Xianwen Liang.

**Supervision:** Xianwen Liang.

**Validation:** Xianwen Liang.

**Writing – original draft:** Hui Liu.
